# Links between ease of use, perceived usefulness and attitudes towards technology in older people in university: A structural equation modelling approach

**DOI:** 10.1007/s10639-022-11292-1

**Published:** 2022-08-17

**Authors:** Marta Liesa-Orús, Cecilia Latorre-Cosculluela, Verónica Sierra-Sánchez, Sandra Vázquez-Toledo

**Affiliations:** 1 Department of Educational Sciences, Faculty of Human Sciences and Education, University of Zaragoza, Valentín Carderera, 4, 22003 Huesca, Spain; 2 Department of Educational Sciences, Faculty of Education, University of Zaragoza, Pedro Cerbuna, 12, 50005 Zaragoza, Spain

**Keywords:** Technology, Intention of use, Attitudes, Ease of use, Perceived usefulness, Older people

## Abstract

Technological resources have the potential to improve the quality of life in a context in which social pressure for the use of these tools is increasing. In this sense, the adoption of technological resources by the elderly is a highly complex issue because numerous and varied factors are involved. Precisely for this reason, this study aims to analyze the effects that exist between a series of dimensions related to the perception of older people regarding the ease of use, the perceived usefulness of technological tools, attitudes towards technology and their intention to use them in everyday life. To do this, 415 adults (M = 66.27 years) enrolled in a program at the University of Experience in the Spanish context completed an online questionnaire. The application of a Structural Equations Model for data analysis highlights that the perceived ease of use of the technology has a positive effect on the perceived usefulness of these resources. Similarly, an indirect effect of the perceived usefulness of technology on the intention to use these resources is observed through the manifestation of positive attitudes towards the use of digital tools.

## Introduction

Healthy ageing, equitable access to learning and quality of life for older people are issues that feature prominently on the global political agenda for 2030, as well as in twenty-first century society. Given the importance of education and relevant role that technology occupies today, two of the Sustainable Development Goals established by the member states of the United Nations, refer to the right of older people to education throughout their entire lives and to equal access to all forms of learning, including digital learning, without discrimination based on age or any other reason. For this reason, the World Health Organization, together with other associations, is implementing strategies to enhance the role of institutions in digital literacy for the social inclusion of older adults. This decision is supported by different investigations (Block et al., 2019; Canedo-García et al., 2022; Carenzio et al., 2021; Hänninen et al., 2020; Olsson & Viscovi, 2018; Tirado-Morueta et al., 2021) and they show that the increase in age increases the need for institutional support and direct teaching to achieve digital literacy in these populations.

However, recent studies carried out on this adult population (Macedo, 2017; Wallcook et al., 2019) show that the elderly continue to be the most vulnerable population in this digitization process. In this sense, the digital divide characterized by the difficulty of access, use or impact of ICDTs on the elderly alludes to a key aspect of twenty-first century society. In this regard, ICDTs offer a series of opportunities, but also some barriers, especially in sectors of the population such as the elderly (Barrantes & Cozzubo, 2017). However, it is evident that today digital technologies are essential and people of all ages require their use to carry out daily activities and participate actively in society. For this reason, it is important to facilitate their access and use in the population and thus prevent them from feeling excluded from it (Barrantes & Cozzubo, 2017; Canedo-García et al., 2022; Capis et al., 2018).

Along the same lines, this growing digital divide has an implicit social character that has been addressed in other research (Barrantes & Cozzubo, 2017; Burnes et al., 2019; Canedo-García et al., 2022; Guner & Acarturk, 2020; Lee & Kim, 2019; Yasunaga et al., 2016). In them, the possibilities offered by relationships between young people and older people to promote the use of ICDT have been highlighted. Likewise, intervention programs have been designed and applied in which people of different ages, young and old, carry out activities together. This type of program promotes the use of ICDT among the elderly and generates great benefits for all the people involved. In this way, a solution is offered to the resistance that older people encounter in the use of these tools due to their insecurity and lack of previous experiences (Tirado-Morueta et al., 2021).

After a thorough review of the existing literature to date and with the aim of delving into the topic addressed, a variety of theoretical models have been found that attempt to explain the adoption of ICDTs among older adults. Among these models, the Theory of Reasoned Action (TRA) proposed by Fishbein and Ajzen (1975) stands out, which defends that people engage in behaviors in which they consider that they will achieve the expected results. And, later and as an adaptation to the aforementioned model, the Technology Acceptance Model (TAM) proposed by Davis (1989) appears, which considers that the perceived ease of use and the perceived usefulness of ICDTs are determining factors in the intention to use ICDTs. (Francis et al., 2019). It is one of the most widely accepted models among the scientific community. To date, different studies have been found (Guner & Acarturk, 2020; Ma et al., 2016; Mariano et al., 2021; Vaziri et al., 2020) that allude to the ease of use, the perceived usefulness of the ICDT and other aspects related to the attitude towards ICDT as factors that influence the relationship and acceptance of ICDT by older people, based on the TAM technological acceptance model.

Among the aspects mentioned in the theoretical model, the perceived usefulness is one of the influential factors in the acceptance of ICDTs in this group, according to several authors (Tirado-Morueta et al., 2021; Canedo-García et al., 2022). In addition, the recent events that have occurred in our society after the outbreak of Covid-19 have highlighted the usefulness of ICDTs in older people as a means of avoiding obstracism, increasing social contact, as well as carrying out routine tasks (Jiménez et al., 2021). However, the perceived usefulness of ICDT by older people depends on many factors. As shown in several studies (Alonso et al., 2021; Basakha et al., 2019; Carenzio et al., 2021; Nimrod, 2017), this perception is negative when the use made about them is scarce. However, there are other studies (Hur, 2016) that show that older people perceive the use of technologies as important for their daily life and feel safe in their use.

Regarding the attitude of people towards ICDTs, these can be influenced by age, training and the use made of them (Halmdienst et al., 2019; Keränen et al., 2017; Seifert & Charness, 2022; Tirado-Morueta et al., 2021; Vroman et al., 2015; Zambianchi et al., 2019). As these studies show, older age makes it more difficult to use them, so their attitude towards them is more negative. However, a higher educational level in older people promotes a more positive attitude towards the use of ICDTs (Zambianchi et al., 2019). On the other hand, a study by the World Health Organization concludes that its frequent use is strongly associated with the development of digital skills (Tirado-Morueta et al., 2021).

Considering the theoretical references available up to now regarding the ease of use and the perceived usefulness of technological tools in populations of older people, this study has attempted to provide a current view of their relationship with other perceptions such as such as the attitude towards ICDTs and their intention to use them. In addition, the study aims to support the need to assess the importance of these factors in the learning process of the elderly population who have decided to study for a university degree. More specifically, the main purpose of this research is to analyze the relationships between different factors that determine the intention of older people to use technology in their daily lives. The possible link between the perceived ease of use of ICDTs and their usefulness, and the intention to make regular use of these tools has been evaluated. Similarly, the existence of a mediating effect of predispositional attitudes towards technology has been investigated on the relationship between the perceived usefulness of these tools and their intention to use them.

## Method

### Participants

A sample of 415 older adults who are enrolled in different courses of the so-called "University of Experience" from the Spanish Higher Education context participated in the study. It is a university program aimed at people over 55 years of age who are looking for lifelong learning. The program is taught by university professors and deals with the study of different humanistic, historical, scientific and artistic contexts from a current perspective. The sampling procedure began by establishing contact via email with the director of this program at a Spanish university. In a second stage, it was this representative person who distributed the web link that led to the data collection platform among the group of older people. At the end of the process, a total of 415 valid questionnaires were received. To be considered admissible, at least 75% of the questions included had to have been completed. Thus, the contextual variables and personal characteristics of the sample are shown in Table 1.

**Table 1:** Sociodemographic characteristics of the sample (*N* = 415)

Variables	*n*	% of the sample
Academic course		
*1°*	131	31.6
*2°*	86	20.72
*3°*	47	11.32
*4°*	32	7.71
*5°*	38	9.16
*6°*	28	6.75
*7°*	53	12.74
Sex		
*Man*	181	43.61
*Woman*	234	56.39
Age (M = 66,27 years; Sd = 6.36)		
55 to 60 years old	54	13.02
From 61 to 65 years	136	32.77
From 66 to 70 years	140	33.73
71 years and older	85	20.48
Total	415	100

Of all the participants, 56.39% were represented by women. The age range of most of the sample was between 61 and 70 years (66.5%). On the other hand, approximately 13% of the sample were between 55 and 60 years old, while 20% were over 70 years old. Considered as a whole, the average age of the sample is around 66 years old, considering that the age range is between 55 and 84 years old. Regarding the course in which these elderly people were enrolled, a predominance of the first two (first course: 31.6%; second course: 20.72%, respectively) and the last of them (seventh course: 12.74%) was observed.

### Definition of variables and instrument

Once the available literature on the object of study had been reviewed, each of the constructs whose relationships were to be analysed later were considered as a starting point. First, the measurement indicators of each of the constructs were conceptually defined. Specifically, some of the already validated subscales contained in the study by Guner and Acarturk (2020) were adapted. In turn, these authors were based on other studies for the creation and validation of the data collection instrument (Davis, 1989; Park et al., 2013). These dimensions were measured from perceptual data from the group of older people participating in the research.

To determine the content validity of the instrument, an expert judgment was planned. Four judges participated in it, all of them professionals from the university academic context from different disciplines: education, research methods in education and behavioral sciences. Each one of them established the adjustment that each indicator presented according to the dimension in which it was included. Similarly, they also valued the wording of the items. Finally, the confidentiality of the data was preserved by guaranteeing the completion of the questionnaires anonymously.

First, the perceived ease of use of the technology (EASE) is the first variable to be analyzed. This construct is defined as a person's perception of the ease of a particular system or technology when used within a defined context (Davis, 1989). In this dimension, a total of 3 indicators were included, among which simplicity in learning to use ICDT and ease of use were considered.

Second, 4 indicators were arranged to measure the perceived usefulness of the technology (US). This construct is understood as the subjective opinion that a person has about the use of a system or technology in a specific context (Davis, 1989). The indicators included the following contents: the usefulness of ICDT to carry out daily activities quickly, the improvement of efficiency in the performance of daily activities thanks to technology, the facility to successfully complete daily activities with the use of ICDT and, finally, the general perception of the usefulness of technology.

The intention to use technology (INT), understood as a person's perception of the possibility of using a certain technology (Venkatesh et al., 2003), was defined with 4 indicators. Among them, the intention to use more technological tools in daily life and the support of ICDT for learning planning and collaborating with classmates was valued.

The last variable proposed and analyzed in this study refers to the attitudes of predisposition towards the use of technology (ATT). Attitudes are defined as the feeling (positive or negative) of a person towards the use of a particular system (Venkatesh et al., 2003). A total of 9 indicators were included for the definition of this dimension. The perception of enrichment thanks to the use of ICDT, the need to constantly update in the use of technology, the increase in motivation with these tools, the perception of improvement in the quality of education and the promotion of creativity and imagination were included.

For all the dimensions of the study, the items were measured on a Likert-type assessment scale of 0–10 because it is the most commonly used measurement and evaluation scale in the Spanish educational system (Bisquerra & Pérez-Escoda, 2015). The number “0” indicated “totally disagree” with the indicator, while “10” meant “totally agree”. The participants responded in this second part to 20 indicators.

### Data analysis

In response to the objective of the study initially proposed, in a first phase the descriptive statistics (in terms of means and standard deviations) of the indicators that made up each of the dimensions related to technology were analysed: perceived ease of use, perceived usefulness, the intention to use and, finally, the attitudes towards these technological tools. These analyses were performed with the SPSS version 22.0 server.

For the second phase, the data analysis methodology corresponds to the Structural Equation Models with latent variables (SEM-LV). This approach makes it possible to estimate and evaluate measurement models and structural models based on robust statistics. Under this approach, the researcher includes the information a priori and can subsequently assess its relevance or not (Bentler, 2006). In addition, this methodology allows reformulating the modelling that was initially included (Bollen, 1989). The models of this study were estimated with the MPLUS version 7.4 software using robust maximum likelihood (Muthén & Muthén, 1998-2007). The modelling was carried out considering the robust statistics and goodness-of-fit indices to multivariate nonnormality. The corrections proposed by Satorra and Bentler (1994) for the goodness-of-fit statistics and for the estimates of the standard errors of the estimated parameters were fixed.

At first, the measurement model of the analysis dimensions of the study was analysed. For this, a confirmatory factor analysis was carried out with the indicators of the theoretical dimensions. Each of these dimensions has the characteristic that they cannot be measured directly. Therefore, they must be deduced according to the observed indicators (items). Factor scores and explained variance coefficients were estimated for ease of use, perceived usefulness, intention to use, and attitudes toward technology. The standardized factor loadings of the observed indicators are considered as evidence of the reliability of the latent variables. These factor loadings must be statistically significant. As a result, their explained variance coefficients should indicate a clear relationship with the underlying factor (or latent variable) (R^***2***^ > 0.50). Precision measures of latent variables were assessed using the AVE and the CRC. For the AVE coefficient of Fornell and Larcker (1981) a minimum reference value of 0.50 was taken, while a minimum value of 0.70 is considered adequate for the omega coefficient (CRC) of McDonald (1985).

After this, the structural model with latent variables was evaluated taking as reference different statistics and indices of global goodness of fit of the proposed model. Specifically, the robust Satorra-Bentler χ^***2***^ statistic was specified for the model. This statistic is determined by the size of the sample and the model. Therefore, the larger the sample of participants, the higher the chi square. Consequently, it is more likely to be significant (Hu & Bentler, 1999). The RMSEA, the SRMR and the CFI were also used. An RMSEA value in a range between 0.05 and 0.10 reflects a proper fit (Hu & Bentler, 1999). On the other hand, the values for the SRMR can vary from 0 to 1, although those models with a more adequate adjustment obtain values below 0.05. Even with this, a value as high as 0.08 could be considered within the limits of what is acceptable (Hair et al., 2006). Finally, a CFI value greater than or equal to 0.95 would show a good fit (Hooper et al., 2008).

In light of the theoretical background exposed, it was proposed that the relationship between the variables should satisfy the following conditions: first, the perceived ease of use has a direct effect on the perceived usefulness of the technology. Second, this perceived usefulness has a direct influence on the intention to use ICDTs. And finally, the perceived usefulness also has an indirect effect on the intention to use, as positive attitudes towards technological tools are increased. If no significant direct effects are found between perceived usefulness and intention to use when the mediating variable (attitudes towards ICDTs) is present, it will be determined that the influence of this perceived usefulness is "completely" mediated by the mediating variable. On the other hand, and if the perceived usefulness significantly influences the intention to use ICDT even in the presence of the mediating variable, it will be concluded that the effects of the perceived usefulness are "partially" mediated (Baron & Kenny, 1986).

## Results

### Descriptive statistics

Table 2 presents a series of preliminary results. In the first place, it is observed that the perceived ease of use of the technology fluctuates at intermediate levels on a scale of 0 to 10. The indicator valued with the highest score refers to the perceived simplicity in the use of the technology (M = 6.68, Sd = 2.21), followed by the general perception that ICDTs are easy to use (M = 6.17, Sd = 2.30). Regarding the perceived usefulness of the technology, the average scores of the items have been slightly above the average. The belief that ICDTs are useful in people's daily lives has received the highest average rating (M = 7.52, Sd = 2.24). The perception that technology facilitates the successful performance of daily activities was the lowest scored indicator (M = 6.31, Sd = 2.72).

**Table 2:** Descriptive statistics and empirical measurement model

	M (Sd)	EASE	US	INT	ATT	R^*2*^	EASE	US	INT	ATT
EASE1. Learning to use ICDTs would be easy for me	6.68 (2.21)	.95				.87				
EASE2. It would be easy for me to make ICDTs do what I want them to do	6.13 (2.24)	.83				.69				
EASE3. I think ICDTs are easy to use	6.17 (2.30)	.92				.85				
US1. The use of ICDTs allows me to carry out my daily activities more quickly	7.00 (2.57)		.90			.81				
US2. The use of ICDTs improves my efficiency in the performance of daily activities	6.48 (2.72)		.96			.92				
US3. The use of ICDTs makes it easier for me to carry out my daily activities successfully	6.31 (2.72)		.95			.90				
US4. I believe that ICDTs are useful in people's daily lives	7.52 (2.24)		.78			.61				
INT1. I intend to use ICDTs more in my daily life	5.10 (2.46)			.63		.40				
INT2. I will use ICDTs to support my learning	6.80 (2.27)			.86		.74				
INT3. I intend to use ICDTs to plan my learning	6.16 (2.47)			.89		.79				
INT4. I intend to use ICDTs to work collaboratively with my colleagues	5.98 (2.68)			.81		.66				
ATT1. I think using ICDTs is a good idea	7.39 (2.14)				.87	.76				
ATT2. I think that using ICDTs can be fun	6.53 (2.39)				.81	.66				
ATT3. I like to use ICDTs	6.79 (2.54)				.87	.76				
ATT4. With ICDTs I can learn anywhere and at any time	7.67 (2.26)				.84	.71				
ATT5. Permanent updating in ICDTs is essential	8.51 (1.77)				.75	.56				
ATT6. ICDTs enrich the teaching–learning process	8.16 (1.78)				.83	.69				
ATT7. ICDTs encourage creativity and imagination	7.01 (2.72)				.78	.61				
ATT8. The use of ICDTs increases people's motivation	7.15 (2.18)				.81	.66				
ATT9. ICDTs improve the quality of education	7.25 (2.12)				.80	.64				
EASE							1.00			
US							.59	1.00		
INT							.52	.61	1.00	
ATT							.74	.75	.74	1.00
*α*		.92	.94	.87	.95					
*CRC*		.90	.90	.80	.82					
*AVE*		.80	.81	.65	.67					

χ^2^ [164] = 802.777 RMSEA = .09 CFI = .85 SRMR = .07

In general terms, average values ​​slightly higher than 5 are observed in the intention to use the technology of the group of older people in the sample. Specifically, the intention to use ICDTs to support the learning process received the highest mean score (M = 6.80, Sd = 2.27), while the lowest score was obtained in the intention to use ICDTs more frequently in daily life (M = 5.10, Sd = 2.46). Regarding the descriptive statistics of attitudes towards technological tools, the means of the indicators of this dimension have been significantly high, all of them exceeding the number of 6.50. In this way, older people consider permanent updating in the use of technology essential (M = 8.51, Sd = 1.77) and the enrichment of the teaching–learning process thanks to these resources (M = 8.16, Sd = 1.78). The mean score decreases when reference is made to having fun with the use of ICDT (M = 6.53, Sd = 2.39) and liking for using technology (M = 6.79, Sd = 2.54).

### Validation of the measurement model

To estimate the validity of the proposed measurement structures, a confirmatory factor analysis corresponding to the measurement model was carried out (Table 2). The statistics and goodness-of-fit indices of these measurement models made it possible not to reject these structures. Thus, the confirmatory analysis presents a reasonable fit (χ^2^ [164] = 802.777, RMSEA = 0.09, SRMR = 0.07, CFI = 0.85). Considering the estimates of the parameters, there is evidence of reliability and convergent validity. The set of factor loadings are significant and the explained variance coefficients (R^2^) exceed 0.40. Finally, the reliability coefficients of the latent variables exceed the minimum cut-off points, while the minimum value of AVE is 0.67 and that of CRC is 0.80. Therefore, the existence of four theoretical constructs in the proposed model is confirmed: ease of use, perceived usefulness, intention to use and positive attitudes towards the use of technology.

#### SEM analysis and mediation analysis

Once the dimensional structure of the latent variables had been evaluated, the effects hypothesized in the theoretical model were analysed. Table 3 specifies the results of the proposed model. Being reasonable enough, its goodness-of-fit statistics allow us to consider that the global model is adjusted (χ^2^ [165] = 805.498, RMSEA = 0.09, SRMR = 0.07, and CFI = 0.85).

**Table 3:** Results of the Structural Model

	EASE	US	ATT	INT
*DIRECT EFFECTS*				
*EASE*		.59***		
*US*			.48***	.11
*ATT*				.66***
*INDIRECT EFFECTS*				
*US*				.32***
*R*^*2*^		.35	.70	.56
*Goodness of fit:*	χ^2^ [165] = 805.498 RMSEA = .09 CFI = .85 SRMR = .07			

First, the positive and statistically significant effect that ease of use exerts on the perceived usefulness of technological tools is observed (EASE: β = 0.59, *p* value < 0.000). This perceived usefulness is not directly associated with the intention to use ICDTs (US: β = 0.11, *p* value > 0.05). However, the perceived usefulness of technology has a direct and significant effect on positive attitudes towards these resources (β = 0.48, *p* value < 0.000). Similarly, a direct and significant effect of favourable attitudes towards ICDTs on the intention to use is also observed (β = 0.66, *p* value < 0.000). Interpreting this set of relationships, the proposed model translates into the fact that the more user-friendly the technology is perceived to be, the greater the perceived usefulness of these tools. At the same time, a high perceived usefulness of ICDTs entails more favourable attitudes towards their use and, in turn, the intention to use them will be greater.

**Fig. 1:**
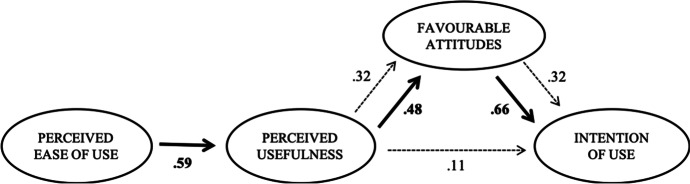
Diagram of results and effects of dimensions on technology

In parallel, the effects of the predictor variables on the outcome variables were calculated. The results show a mediating effect of positive attitudes towards ICDTs on the relationship between the perceived usefulness and the intention to use the technology (β = 0.32, *p* value < 0.000). Therefore, the effect between both variables (perceived usefulness and intention to use) will be increased if the first one translates into more favourable attitudes towards technology. By including the mediating variable (attitudes towards ICDTs) in the model, an effect of the perceived usefulness on the intention to use originates. Consequently, it would be considered a total mediation model. Figure 1 illustrates a visual synthesis of both direct and indirect effects of the global model under test.

## Discussion

The objective of this study focused on examining the relationships between different factors that could exert some kind of influence on the intention to use ICDT in older people. In this way, the findings found in this study show the relationships between the different variables analyzed, as well as reflect the factors that can influence the intention to use ICDT by older people who have decided to enroll in the "University of the experience". Among these variables are the perceived ease of use of the technology, its perceived usefulness and the attitude towards technologies as factors that can influence or determine the intention to use ICDTs in this population group. These factors have been considered after a previous review of the existing literature to date on the subject addressed in this study. In this way, the results obtained after carrying out the corresponding statistical analysis show the existence of positive and significant effects between the factors addressed in this research. Likewise, it is worth mentioning that among the diversity of factors that can influence the intention to use ICDT by older people, the factors studied in this model exert an influence on it.

In this sense, after estimating the measurement and mediation models, the results obtained provide a set of relevant conclusions for the context in which this research unfolds. In this way, the relationships between the factors addressed in this study have defined a research model that reinforces the Technology Acceptance Model (TAM) proposed by Davis (1989) as the basis for understanding the adoption and use of technology among older adults. More specifically, the results provided by the sample participating in this study affirm that the ease of use perceived by the elderly in relation to ICDTs directly affects their perceived usefulness by this group of the population, coinciding with those exposed. by other authors (Al-Maroof et al., 2020; Davis, 1989; Guner & Acarturk, 2020; Mitzner et al., 2010) in their research carried out under the same model. Along the same lines, the study carried out by Chen and Chan (2011), based on the STAM model, concludes that both the perceived ease of use and the perceived usefulness of ICDTs directly influence the adoption of ICDTs. However, other authors (Kuerbis et al., 2017) indicate that the perceived usefulness of ICDTs depends on the degree of awareness that older people have about the benefits that these tools can bring to their daily lives. day and depending on it, they will have a greater or lesser intention of use.

Occasionally, circumstances allow us to highlight the opportunities and facilities that ICDTs offer in certain aspects of our lives, as has recently been shown in our society during the COVID-19 pandemic. This situation has highlighted the opportunities that ICDTs offer on a day-to-day basis for the entire population, but especially to contribute to the participation of older people in our society, maintain personal relationships, be informed, in short, favor their intention to use when knowing its possibilities (Al-Maroof et al., 2020; Khosravi et al., 2016). The importance of ICDTs in the lives of older people is reinforced by studies in which research has been carried out on the possibilities they offer as support and accompaniment in their day-to-day life to avoid the isolation of this group of the population. As stated by Sidner et al. (2018), in a study carried out on the help that a virtual agent or robot can offer elderly people to avoid their isolation.

Likewise, following the model proposed in this study, the intention to use ICDTs in older people is determined based on their perceived usefulness; however, a positive attitude or predisposition is included as a mediating effect between both variables. towards the use of ICDT. In this sense, Zambianchi et al. (2019) stress that older people who have more positive attitudes towards ICDTs are those who make more frequent use of them. In contrast, in the study carried out by Pargaonkar et al. (2019) they found that the factors that affect the use of technology and the attitude towards technology in older people are awareness of ICDT, the perceived importance of technology in their life, the willingness to learn and ask for help, self-efficacy, the willingness to invest time and money, as well as the security and sense of enjoyment perceived in the use of ICDT.

In light of the results presented, and as stated by other authors (Chopik et al., 2017; Czaja et al., 2018; Macedo, 2017; Marston et al., 2019; Rogers & Mitzner, 2017; Tyler et al., 2020), the integration of ICDTs in the lives of older people offers them great opportunities for learning, as well as social participation. In short, ICDTs can contribute to improving the quality of life of the elderly (Menéndez et al., 2020) and this is confirmed by themselves in other studies carried out in recent years (Oliver et al., 2017; Rubenson, 2018).

## Conclusions

The impact of technology on our society is enormous, it is clear that it has permeated all areas of our daily lives. However, the use we make of technology is very varied and on many occasions, it becomes a challenge and sometimes even a barrier, especially in the elderly population. Likewise, this tendency to use technology does not guarantee that its use will be accepted or that it will be generalized. That is why this research sought to know what factors condition the use of technologies in this population.

Considering the results of this research and coinciding with the findings found in other studies, it is evident that there are different factors that contribute to significantly explain the use that older adults make of ICDT, such as the perception of ease of use, usefulness perception, intention to use, and attitudes toward technology (Hong et al., 2013; Wang et al., 2011).

With all of the above, it has been possible to show that the perception of usefulness influences the attitude and this, in turn, influences the intention to use. Likewise, the greater the perceived ease of use, the greater the usefulness is considered. Therefore, the hypotheses raised in this research have been demonstrated and the Technology Acceptance Model (TAM) has been ratified.

An important aspect to highlight is that although the digital transformation is advancing, inequalities in the use of technology in this population continue to exist (Seifert, 2021). In this regard, the study carried out by Alonso et al. (2021) reflected that the use and acquisition of technologies by older adults is not homogeneous. However, ICDTs are a great opportunity that can improve the quality of life and increase the personal well-being of this population (Hill et al., 2015; Tyler et al., 2020). In this way, technological acceptance and the incorporation of the use of ICDT in daily activities should be an objective to be achieved. In this sense, an older adult empowered in its use and with technological skills will be able to use them optimally. This is where techno-digital literacy plays an essential role. If older adults consider that technologies are easy to use because they feel competent, they will be motivated to use them and will overcome some of the main barriers that influence negative attitudes and hinder the process of adopting ICDTs, such as fear, anxiety, lack of motivation and interest (Pargaonkar et al., 2019; Menéndez et al., 2020). For this work, it is considered necessary to adapt the training actions in relation to technology and older adults based on different user profiles, with different realities, needs and interests. In this way, training programs adapted to the different profiles would be developed, considering their trajectories and previous experiences.

In short, it is about promoting e-inclusion programs to improve digital competence in older adults and minimizing the digital divide with the aim of avoiding the risk of digital and therefore social exclusion (Andreasson, 2015), given that technologies currently constitute a growing means of participation and communication in our society.

Regarding the limitations of this research, it would be necessary to contrast the results of this study in a sample of older adults from other contexts, other than university, in order to analyze all these factors in depth. And finally, with regard to future lines of research and taking into consideration what is stated in this work, we agree with Lee and Maher (2021) that it would be necessary to carry out studies that delve into the distinction between the initial commitment, usability and needs.. As well as verifying which factors directly affect older adults and increase their level of commitment to technology, in this case, it would be interesting to analyze, among other factors, their professional career, previous experience, ease of use, family support and access to technology.

## Data Availability

The datasets generated and/or analysed during the current study are available in the Google Drive repository: [https://drive.google.com/drive/folders/1SopBXkD7zeRrC3f1rABOD3xxIggMWa5k?usp=sharing].
